# 

**Published:** 1863-10

**Authors:** 


### CONTENTS.

ORIGINAL ESSAYS AND COMMUNICATIONS.

Epulis. By Leon F. Harvey.......... 417
Treatment of Dental Pulp. By Geo. B Snow.......... 425
Anomalous Cases. By Wm. H. Atkinson, M. D.,.......... 430

CORRESPONDENCE.

Dental Matters in Iowa.......... 435

SELECTIONS.

Effects of Tobacco Smoking.......... 442
Dangerous Forms of Quackery,.......... 446
Absorptive Power of the Skin.......... 448
Red Vulcanile as a Base for Artificial Teeth.......... 449
On an Improved Mode of Using Refrigeration as an Anaesthetic and as a Remedy.......... 451
Diathesis.......... 454
The Moisture in the Air.......... 458

EDITORIAL.

Dental Colleges.......... 460
The Mallet.......... 462
The People's Journal.......... 462

